# The role of iron in the skin and cutaneous wound healing

**DOI:** 10.3389/fphar.2014.00156

**Published:** 2014-07-10

**Authors:** Josephine A. Wright, Toby Richards, Surjit K. S. Srai

**Affiliations:** ^1^Division of Surgery and Interventional Science, University College London, University College & Royal Free HospitalsLondon, UK; ^2^Department of Structural and Molecular Biology, Division of Biosciences, University College LondonLondon, UK

**Keywords:** iron, skin, wound-healing, ultraviolet, iron chelating agents

## Abstract

In this review article we discuss current knowledge about iron in the skin and the cutaneous wound healing process. Iron plays a key role in both oxidative stress and photo-induced skin damage. The main causes of oxidative stress in the skin include reactive oxygen species (ROS) generated in the skin by ultraviolet (UVA) 320–400 nm portion of the UVA spectrum and biologically available iron. We also discuss the relationships between iron deficiency, anemia and cutaneous wound healing. Studies looking at this fall into two distinct groups. Early studies investigated the effect of anemia on wound healing using a variety of experimental methodology to establish anemia or iron deficiency and focused on wound-strength rather than effect on macroscopic healing or re-epithelialization. More recent animal studies have investigated novel treatments aimed at correcting the effects of systemic iron deficiency and localized iron overload. Iron overload is associated with local cutaneous iron deposition, which has numerous deleterious effects in chronic venous disease and hereditary hemochromatosis. Iron plays a key role in chronic ulceration and conditions such as rheumatoid arthritis (RA) and Lupus Erythematosus are associated with both anemia of chronic disease and dysregulation of local cutaneous iron hemostasis. Iron is a potential therapeutic target in the skin by application of topical iron chelators and novel pharmacological agents, and in delayed cutaneous wound healing by treatment of iron deficiency or underlying systemic inflammation.

## INTRODUCTION

Iron is a vital co-factor for proteins and enzymes involved in energy metabolism, respiration, DNA synthesis, cell cycle arrest and apoptosis. Over the past 10 years, major advances have been made in understanding the genetics of iron metabolism and this has led to identification of a number of new proteins, including hepcidin, an acute phase protein that is the master regulator of iron absorption and utilization, often activated in chronic diseases (Weiss, 2009; Finberg, 2013).

Historically, it has long been known that iron is essential for healthy skin, mucous membranes, hair and nails. Clinical features of iron deficiency include skin pallor, pruritus, and predisposition to skin infection (impetigo, boils and candidiasis), angular chelitis, swollen tongue, fragile nails, kolionychia, and dry brittle hair.

## ROLE OF IRON IN THE SKIN

### NORMAL PHYSIOLOGY OF IRON

**FIGURE 1:**
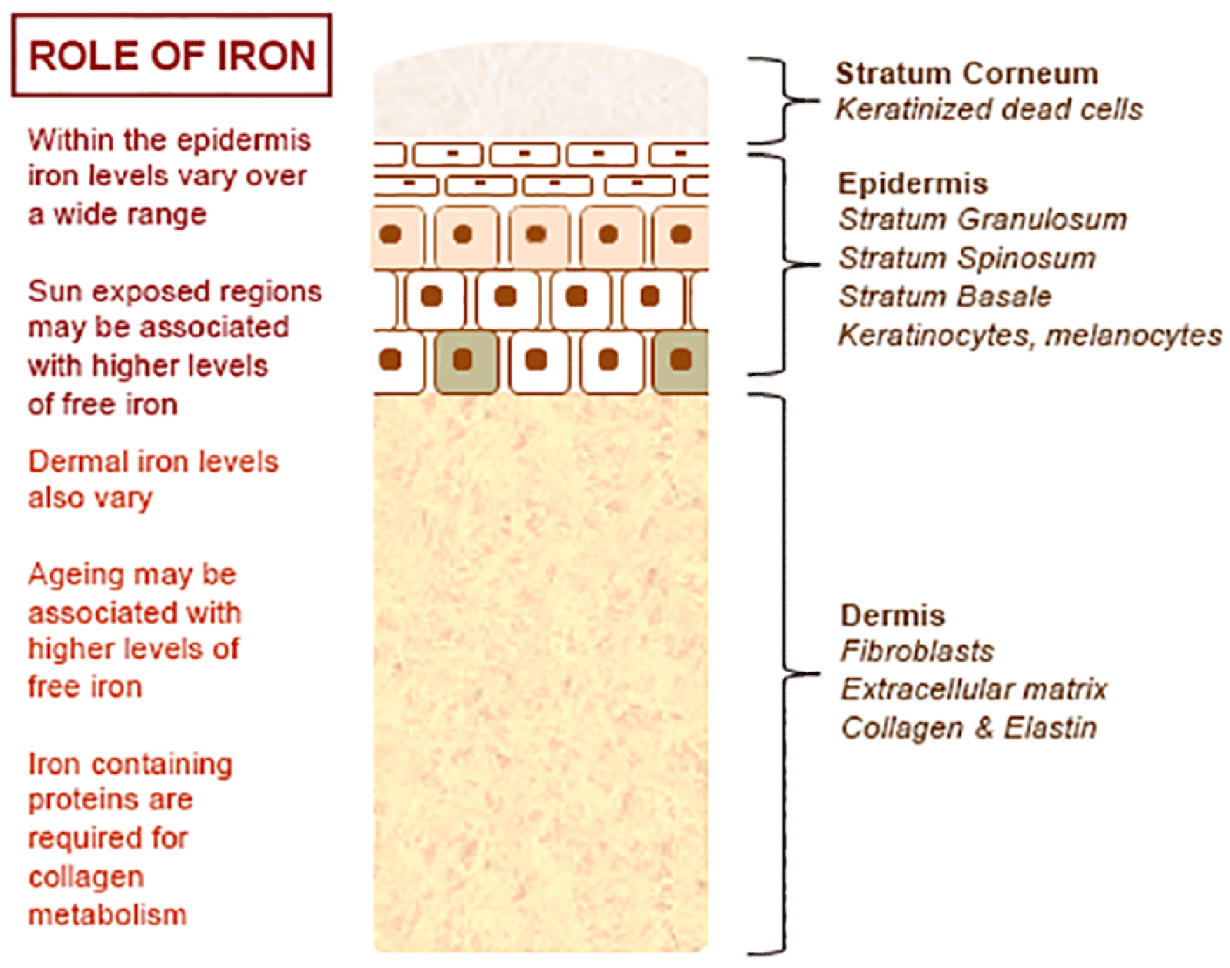
**Role of iron in the skin – an overview**.

**FIGURE 2:**
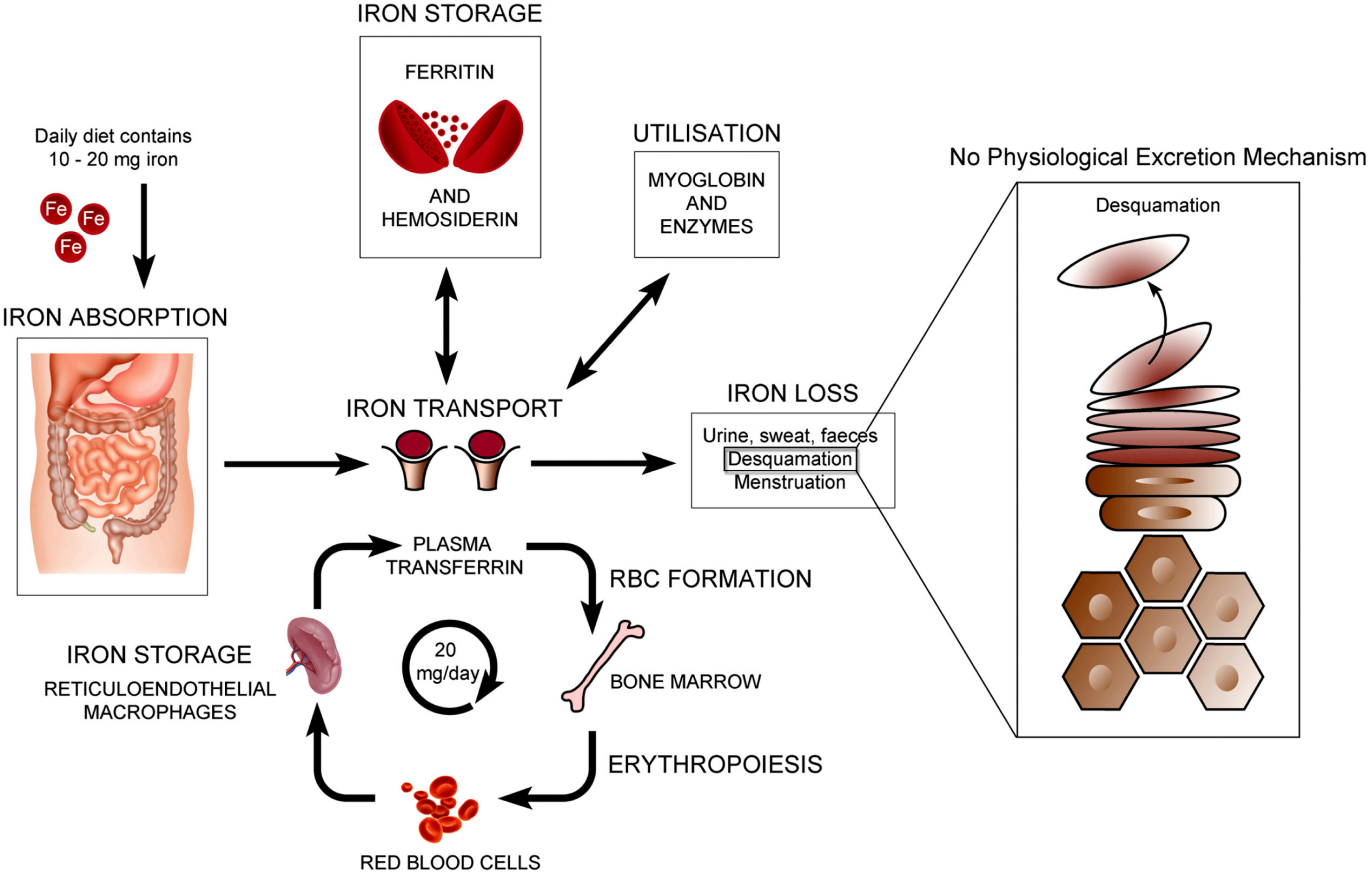
**Normal physiology of iron homeostasis.** Figure adapted to show key iron physiology including iron loss by desquamation (see Milstone et al., 2012).

The normal physiology of iron in the skin is complex and not clearly understood. It is known that iron levels in normal epidermis are thought to vary over a wide range (Molin and Wester, 1976; Kurz et al., 1987). Within normal dermis, iron levels also vary and are thought to increase during the aging process (Leveque et al., 2003). Furthermore, iron-containing proteins have specific function such as the metabolism of collagen by procollagen-proline dioxygenase (Richardson et al., 1996; Polefka et al., 2012; **Figure 1**). Iron is not actively excreted from the body, however the skin is a key organ in iron hemostasis as iron is lost through the skin by desquamation (**Figure 2**). Current theories regarding the underlying mechanisms of desquamation include active dissolution of desmosomes involved in keratinocyte cell–cell adhesion, by hydrolytic protease digestion (Milstone, 2004). Desquamation of keratinocytes is thought to account for 20–25% of absorbed iron that is lost (Jacob et al., 1981). Yet overall, the daily loss of iron by desquamation is approximately 25% that of daily urinary iron excretion (Molin and Wester, 1976). Evidence is emerging from genetic model mouse studies by Milstone et al. (2012) that both loss of iron by desquamation and local changes in epidermal iron metabolism have some role in systemic iron metabolism (these studies investigated three groups of mice: firstly mice overexpressing of HPV16 E7 gene, which causes a threefold increase in epidermal turnover, secondly mice overexpressing the transferrin receptor which causes a three to fourfold increase of epidermal iron in a skin model, and finally a systemic hemochromatosis knockout model crossed with the epidermal iron sink model). Additionally, gender-related differences in iron status may be responsible for the increased longevity of women as compared to men. The relative difference in cell iron levels between the sexes may be of importance both physiologically and in setting of pathophysiological conditions (Pouillot and Polla, 2013).

### IRON, OXIDATIVE STRESS, AND PHOTO-INDUCED DAMAGE

The main causes of oxidative stress in the skin are reactive oxygen species (ROS) generated in the skin by ultraviolet (UVA) 320–400 nm portion of the UVA spectrum. Iron plays a key role in oxidative stress processes, as it is a transition metal, which exists in two stable states, Fe^2+^ (electron donor) and Fe^3+^ (electron acceptor). Intracellular labile iron can undergo redox cycling between its most stable oxidation states (Fe^2+^/Fe^3+^) and react with ROS such as superoxide anion, hydrogen peroxide, giving rise to hydroxyl radicals via the Fenton reaction or superoxide-driven Fenton chemistry (Pelle et al., 2011).

Exposure of skin fibroblasts to UVA can generate ROS that promote oxidative damage in lysosomal, mitrochondrial, nuclear, and plasma membranes. Ultimately loss of plasma membrane integrity together with mitrochondial ATP depletion results in necrotic cell death (Aroun et al., 2012). It is thought that compared with skin fibroblasts, keratinocytes are more resistant to UVA mediated membrane damage and cytotoxicity. In vitro studies have shown that although UVA starts lysosomal damage, ferritin degradation and cytosolic labile iron release in keratinocytes, the absolute level of UVA induced labile iron release is several fold lower than in fibroblasts, suggesting a link between labile iron release and keratinocyte resistance to UVA mediated damage (Zhong et al., 2004).

## ANEMIA, IRON DEFICIENCY, AND CUTANEOUS WOUND HEALING

**FIGURE 3:**
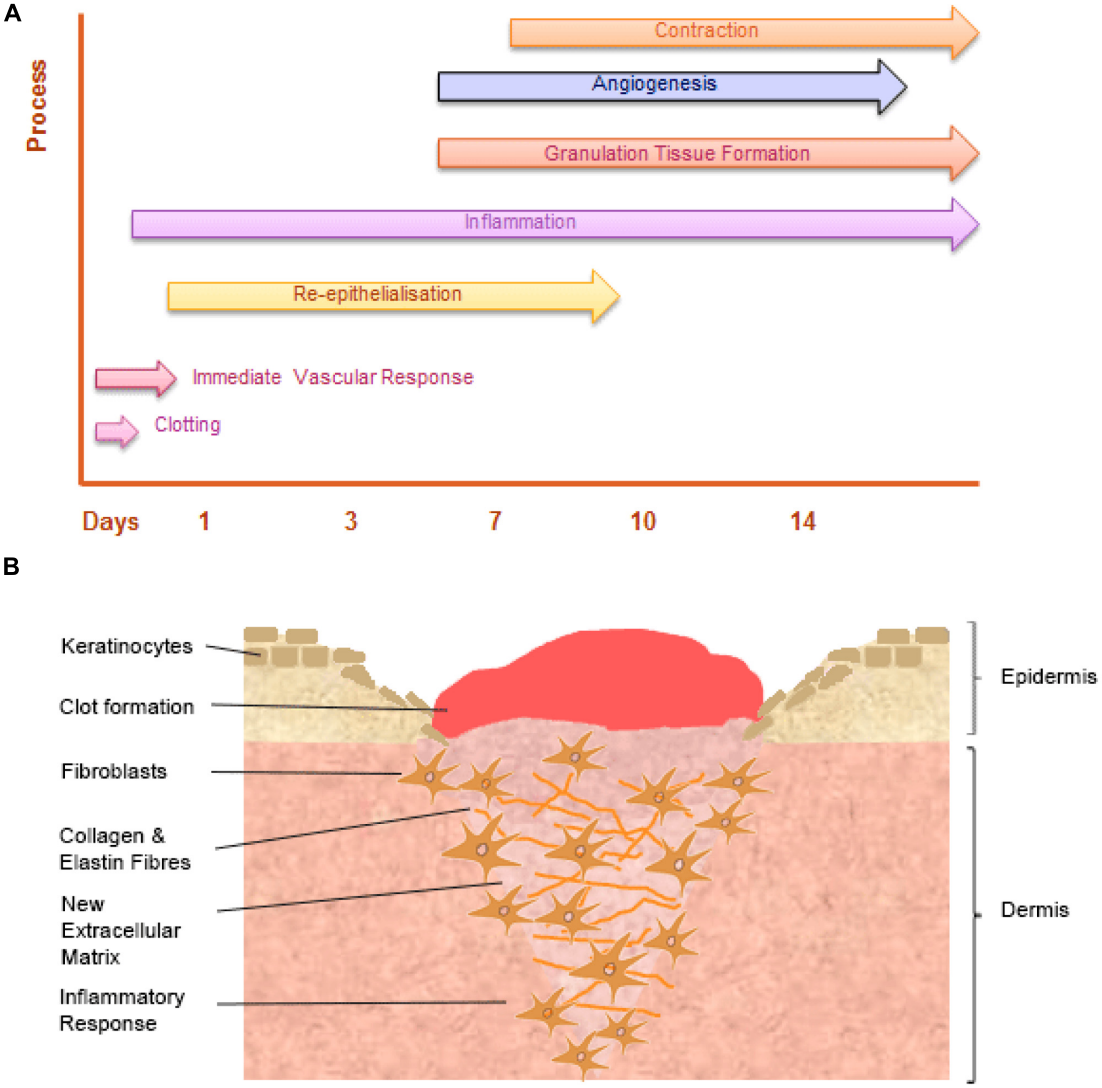
**The temporal stages of cutaneous wound healing and key cell types involved. (A)** Graph illustrating the key temporal stages of wound healing over the 14 days post injury. **(B)** The key cell types involved in the wound healing process.

Wound healing is a dynamic and highly regulated process consisting of cellular, humoral and molecular mechanisms (Reinke and Sorg, 2012). The normal cutaneous wound healing process is a temporal process involving a complex series of overlapping events, which can be divided into key stages including: hemorrhage and fibrin-clot formation, inflammatory response, re-epithelialization, granulation tissue formation, angiogenic response, connective tissue contraction, and remodeling, see **Figure 3**.

## EXPERIMENTAL STUDIES – IRON, ANEMIA, AND WOUND HEALING

In current literature, animal studies fall into two distinct groups. Early studies investigating the effect of anemia on wound healing used a variety of experimental methodology to establish anemia or iron deficiency. They focused on wound strength studies rather than initial macroscopic healing or histological studies of re-epithelialization. More recent studies have investigated novel treatments aimed at correcting the effect of systemic iron deficiency and topical application of iron-chelators to reduce iron at the specific site of inflammation and in particular their effect on pro inflammatory macrophages.

### IN VIVO STUDIES – THE EFFECT OF IRON DEFICIENCY ON WOUND HEALING

Early initial experimental rodent studies used powdered milk diet to establish iron deficiency. Jacobson and Vanprohaska (1965) found that chow-fed control mice showed significantly higher wound breaking-strength than anemic mice that were fed on an iron-free powdered milk diet. Bains et al. (1966) found that young rats fed low iron (powdered milk) diet and subjected to repeated bleeding to produce chronic anemia had weaker wound tensile strength. However, later studies undertaken by Macon and Pories (1971) had contrary findings; iron-deficiency anemia (IDA) had no effect on wound breaking strength. This may reflect the methodological issues when using powdered milk to establish iron deficiency as Waterman et al. (1952) showed that control and anemic rats fed powdered milk had slower wound contraction and reduced wound breaking strength, when compared with animals fed normal chow.

Investigation into the impact of anemia and blood volume on wound healing strength by Sandberg and Zederfeldt (1960) found that replacing blood volume with dextran restored normal wound healing, following acute hemorrhage in a rabbit model. Heughan et al. (1974) found that there was no significant change in wound-fluid oxygen tension (PO_2_) in rabbits made anemic by bleeding and re-transfusing plasma. Additionally, connective tissue weight was greater at lower packed cell volumes, an initial finding that suggested a deleterious effect of hypoxia on collagen synthesis.

Oliveira Sampaio et al. (2013) investigated the effect of delivery of an iron free diet for 15 days in Wistar rats, though histological study of excisional wounds at 7, 14, and 21 days post-healing. LED light caused a significant positive bio-modulation of fibroblastic proliferation in anemic animals, with laser being more effective on increasing proliferation in non-anemic animals. This study did not describe the effect of iron deficiency on the early stages of wound healing (re-epithelialization) or later resolution.

There are various mechanisms by which iron deficiency may impair wound healing. Current evidence favors a key role played by hypoxia. Hypoxia-inducible factor-1 (HIF-1) contributes to all stages of wound healing (through its role in cell migration, cell survival under hypoxic conditions, cell division, growth factor release, and matrix synthesis) and positive regulators of HIF-1, such as prolyl-4-hydroxylase inhibitors, have been shown to be beneficial in enhancing diabetic healing (Hong et al., 2014). Further studies are required to directly answer this question.

Of note, the functional role of iron in the wound healing process has not undergone detailed *in vitro* study. Recent interest in lactoferrin, an iron-binding glycoprotein secreted from glandular epithelial cells, has focused on its role in promoting cutaneous wound healing by enhancing the initial inflammatory phase, and cell proliferation and migration. Takayama and Aoki (2012) found using an in vitro model of wound contraction, lactoferrin promoted fibroblast-mediated collagen gel contraction.

### *IN VIVO* STUDIES – THE EFFECT OF IRON CHELATORS ON CUTANEOUS WOUND HEALING

There is considerable variability in iron chelator structure, mechanism of action and their consequent applications. The most widely used iron chelator used to treat iron over-load is deferoxamine, see **Table 1**. Different iron chelators have been applied in studies using a variety of wound healing models.

**Table 1:** Summary of iron chelators.

Iron Chelator	Summary			
	Structure	Derivative	Mechanisms	Application
**Deferoxamine** *N’*-{5 [Acetyl(hydroxy)amino] pentyl}-*N*-[5-({4-[(5-aminopentyl) (hydroxy)amino]-4-oxobutanoyl}amino)pentyl]-*N*-hydroxysuccinamide	Hexidentate structure comprising multiple carbonyl and hydroxyl groups that donate electrons Fe^3+^, making it chemically inert, by preventing further redox cycling. Chelates iron in a one-to-one ratio	Bacterial siderophore produced by actinobacteria	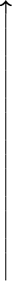	Clinically, the most widely used iron chelator to treat iron over-load. Applied topically to the skin in experimental studies

**Kojic Acid** 5-Hydroxy-2-(hydroxymethyl)-4H-pyran-4-one	Bidentate iron chelator	Various species of *Aspergillus* and *Penicillium* in an anaerobic process	Varying rates of interaction with cellular iron pools in different tissues; effects on plasma iron pools remain incompletely understood	Current applications are cosmetic: “natural” antioxidant and skin lightener
**Deferiprone** 3-hydroxy-1,2-dimethylpyridin-4(1*H*)-one	Bidentate iron chelator	Antibacterial effect	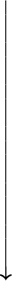	Clinically used for beta-thalassemia major treatment; its use is limited by toxicity (agranulocytosis/ liver failure). Applied topically to the skin in experimental studies.
**Ciclopiroxolamine** 6-cyclohexyl-1-hydroxy-4-methylpyridin-2(1*H*)-one	Lipophilic bidentate iron chelator. Causes loss of function of catalase and peroxidase enzymes	Also classified as a hydroxypyridinone antifungal agent. Further anti-inflammatory properties		Topical treatment of onychomycosis, tinea pedis and corporis.

As can be seen, iron chelators may have antibacterial/antifungal, anti-inflammatory and skin lightening effects (Porter, 2009). These mixed effects limit the testing of experimental hypothesis surrounding role of iron and iron deficiency in cutaneous wound-healing.

Early studies of porcine flap necrosis found that intramuscular injection of deferoxamine decreased the percentage of flap necrosis (Weinstein et al., 1989). This study provided some indirect evidence suggesting that iron chelators have a positive effect on wound healing. Mohammadpour et al. (2013) carried out a wound healing study in Wistar rats, primarily assessing macroscopic wound area calculations at days 4, 8, and 12. They found that topical deferiprone treatment accelerated macroscopic wound healing more than Kojic acid, and on the basis of further DPPH scavenging assay suggested that this was due to its higher antioxidant and iron chelation abilities.

Iron chelation results in increased VEGF and HIF 1-α and positive effect on angiogenesis The effect of iron chelation on granulation tissue formation and angiogenesis has not been demonstrated in cutaneous wound healing studies, although there have been some studies of bone tissue in the context of fracture healing (Phelps et al., 1986; Farberg et al., 2012; Donneys et al., 2013). Localized Deferoxamine injection has been shown to both reverse radiation induced hypovascularity and augment vascularity in pathologic fracture healing.

The incorporation of iron chelators in novel wound dressing for human chronic wound treatment has also been described. Wenk et al. (2001) suggested that in human chronic wounds, wound fluid iron-levels are elevated compared with acute wounds. They developed a novel wound dressing based on deferoxamine coupled cellulose and in vitro assays suggested that this dressing may target iron-driven induction of matrix-degrading metalloproteinase-1 and lipid peroxidation. Taylor et al. (2005) also developed and described successful biomechanical testing of deferoxamine coupled polyurethane net substrates.

### CLINICAL STUDIES – ROLE OF IRON IN HUMAN CUTANEOUS WOUND HEALING

Human studies in patients with anemia have focused on wound strength. These studies have involved small case-series of patients with a variety of acute surgical conditions. Jonsson et al. (1991) performed a study of 33 patients undergoing subcutaneous implantation of ePTFE graft, collagen deposition was directly proportional to wound oxygen tension and measures of perfusion, although the anemia seen in these patients was not fully described. Pavlidis et al. (2001) carried out a retrospective analysis of 89 patients and found that anemia was not associated with laparotomy wound dehiscence. In a study of 35 normovolaemic anemic patients undergoing skin grafting, Agarwal et al. (2003) found there was no difference in wound healing as assessed by mean split-thickness skin graft take.

To date, human studies have not demonstrated the specific effects of iron deficiency and anemia on the histological stages of chronic wound healing. Clinical studies by our group have found an association between diabetic foot ulceration (DFU) severity and hemoglobin (Hb) decline. DFU is a complex condition, characterized by poor wound healing. Over half all severe DFU patients have IDA (Khanbhai et al., 2012). Clinically the anemia is difficult to characterize; a significant proportion of patients have a functional iron deficiency (FID) caused by chronic inflammation and disruption of the normal Hepcidin mediated iron absorption pathways.

## DYSREGULATION OF LOCAL CUTANEOUS IRON HOMEOSTASIS IN CHRONIC LEG ULCERATION

Chronic inflammatory conditions such as rheumatoid arthritis (RA) and Lupus Erythematosus are associated with dysregulation of local cutaneous iron hemostasis.

RA is a progressive inflammatory autoimmune disease, with joint articular and systemic effects including development of ulceration and poor wound healing. The release of cytokines, especially TNF-α, IL-6, and IL-1, causes synovial inflammation. Pro-inflammatory cytokines also promote the development of systemic effects, including production of acute-phase proteins (such as CRP) which in turn may contribute to development dysregulation of iron homeostasis and anemia (Choy, 2012). Indeed, clinical studies of RA patients have reported both iron deficiency anemia and anemia of chronic disease (Bari et al., 2013). Inflammation upregulates the expression of iron-related proteins in the duodenum and monocytes of RA patients (Sukumaran et al., 2013). Evidence for a role for IL-6 signaling in RA is emerging. Isaacs et al. (2013) found that tocilizumab results in an improvement in anemia, reduction in hepcidin/haptoglobin and increase in iron-binding capacity. Approximately 10% of patients with RA develop leg ulceration.

Clinically, RA leg ulcers are typically associated with venous insufficiency, trauma, arterial insufficiency and rarely vasculitis (for review, see Rayner et al., 2009). Further work is needed to look at the effect of IAD in patients with RA and leg ulceration.

Lupus Erythematosus is an autoimmune disorder with diverse clinical manifestation ranging from mild cutaneous disorder to a life-threatening systemic illness (SLE). Some patients suffer from a skin-limited form (with a variety of manifestations including oral ulceration), while in others it evolves into SLE, although this process is not fully understood. A key exogenous trigger to the onset of cutaneous disease activity is exposure to UV radiation. It has been shown that photosensitive patients with cutaneous lesions express anti-Ro/SSA autoantibodies. In vivo studies have demonstrated up-regulation of antigens such as Ro52 in keratinocytes (Oke and Wahren-Herlenius, 2013). This is of some interest; it is possible that iron release in response to UV radiation impairs the function of these antigens, which appear to play a role in negative feedback in response to inflammation.

## DELETERIOUS EFFECTS OF LOCAL CUTANEOUS IRON DEPOSITION

**FIGURE 4:**
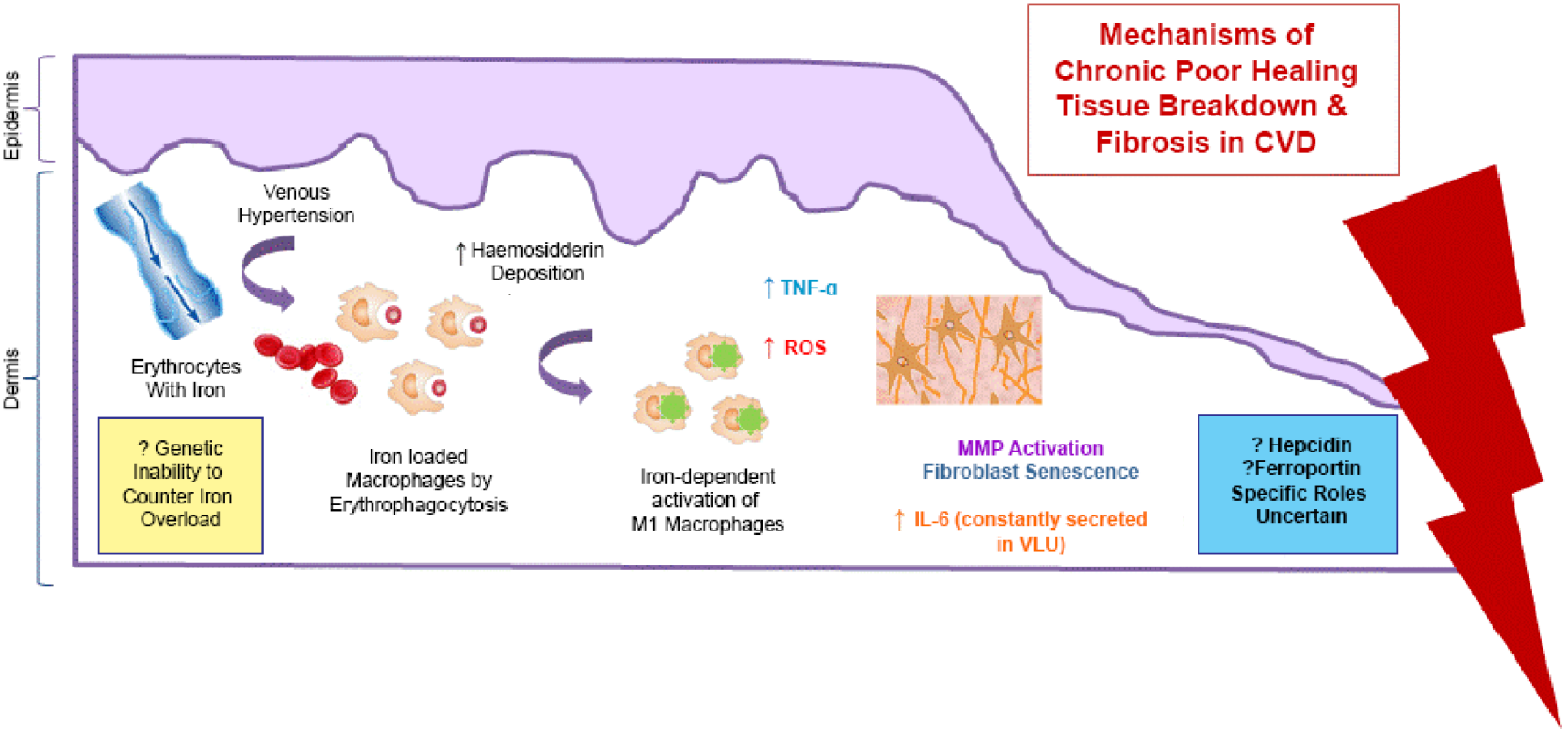
**Mechanisms of the deleterious effects of local cutaneous iron deposition in CVD.** Venous hypertension (characterized by abnormally leaky venous valve) leads to extravasation of erythrocytes with iron. There is increased hemosiderin deposition in the dermis. Macrophages become loaded with iron (by erythrophagocytosis) resulting in unrestrained pro-inflammatory macrophage activation. ROS produced cause a cascade of deleterious reactions and increase oxidative stress. There is further inflammatory response through tumor necrosis factor alpha (TNF-α) and interleukin-6 (IL -6) which is constantly secreted venous leg ulceration (VLU). Dermal fibrosis is the result of matrix metalloproteinase (MMP) activation and fibroblast senescence.

There has been some interest in the role of excess iron stored in the skin as hemosiderin, in the pathophysiology of chronic venous disease (CVD). It is now thought that the severe skin changes (such as lipodermatosclerosis) and leg ulceration associated with CVD happen after iron overload occurs. The mechanisms underlying the deleterious effects of local cutaneous iron deposition in CVD are shown in **Figure 4**.

Recent studies by Sindrilaru et al. (2011) and Sindrilaru (2013) have identified a subset of iron-overloaded inflammatory M1-like macrophages, which are implicated in the pathogenesis of CVD. Hb release from extravasated erythrocytes results in a serum haptoglobin/hemoglobin complex that is then taken up by the macrophages, upon upregulation of the hemoglobin–haptoglobin receptor CD163. In CD163^high^ macrophages, continuous uptake of Hb is thought to be the cause high intracellular concentrations of heme-iron which induce an unrestrained pro-inflammatory macrophage activation. Macrophage iron can be further increased during inflammation by virtue of increased systemic or local hepcidin expression, which leads to reduction in ferroportin, an iron eﬄux protein, resulting in intracellular iron accumulation. Individuals may also be predisposed to CVD disease through a genetic inability to counteract the skin iron overload (Caggiati et al., 2010). Studies have shown that common hemochromatosis gene mutations such as the C282Y mutation significantly increase the risk of ulcer in CVD by almost seven times (Zamboni et al., 2005).

In hereditary hemochromatosis, plasma iron content increases beyond the iron binding capacity of transferrin, although normal erythropoiesis is occurring. Studies using quantitative nuclear microscopy measurements of iron concentration in the epidermis (which is a readily accessible tissue) have shown that skin iron levels reflect the liver iron overload. Interestingly, this technique has been proposed as a clinical tool to enable better informed decisions on when to initiate, change or stop phlebotomy therapy. In both CVD and hereditary hemochromatosis, parenchymal iron deposition leads to activation of metalloproteinases and subsequently fibrosis. Hereditary hemochromotosis has also been used to study the effects of iron on the aging process. Relative iron overload may also have a deleterious effect on normal skin aging, as iron chelators assist “successful” normal skin aging when applied topically (Polla et al., 2003).

Leg ulceration represents one of the main causes of morbidity in sickle cell anemia (SCA). Known risk factors for leg ulcer development in SCA include Hb (≤6 g/dL), lower levels of fetal Hb, hemolysis, raised lactate dehydrogenase (Lobe et al., 1992), infections and inflammation (Cumming et al., 2008). It is likely that in SCA and other disorders causing hemolytic anemia (such as hereditary spherocytosis, thalassemias, and other hemoglobinopathies) the deleterious effects of excessive local cutaneous or macrophage iron deposition play key roles in poor wound healing.

## CONCLUSION

Over recent years there has been some advancement in knowledge about iron in the skin and iron deficiency in cutaneous wound healing. It is clear from studies on pathology of CVD that high iron in macrophages can induce unrestrained proinflammatory macrophage activation. Furthermore in cases of iron deficiency/anemia of inflammation, when serum hepcidin levels are elevated, hepcidin/ferroportin interaction can lead to increased iron concentration in cells particularly macrophage and this could also have a detrimental effect on wound healing. Iron deficiency without inflammation is likely to affect one of the later stages of wound healing such as remodeling. Additional in-depth scientific study of both the underlying pathophysiological mechanisms and role of local cutaneous iron in conditions associated with iron overload and iron deficiency is a priority. Iron is a potential therapeutic target in the skin by application of topical iron chelators and other novel pharmacological agents, and in delayed cutaneous wound healing by treatment of iron deficiency.

## Conflict of Interest Statement

The authors declare that the research was conducted in the absence of any commercial or financial relationships that could be construed as a potential conflict of interest.
